# Axonal regeneration and innervation ratio following supercharged end-to-side nerve transfer

**DOI:** 10.3389/fcell.2025.1513321

**Published:** 2025-02-12

**Authors:** Leopold Harnoncourt, Martin Schmoll, Christopher Festin, Laurenz Pflaum, Markus Breuss, Johanna Klepetko, Dominik C. Dotzauer, Florian J. Jaklin, Udo Maierhofer, Philipp Tratnig-Frankl, Oskar C. Aszmann

**Affiliations:** ^1^ Clinical Laboratory for Bionic Extremity Reconstruction, Department of Plastic, Reconstructive and Aesthetic Surgery, Medical University of Vienna, Vienna, Austria; ^2^ Center for Medical Physics and Biomedical Engineering, Medical University of Vienna, Vienna, Austria; ^3^ Department of Plastic, Reconstructive and Aesthetic Surgery, Medical University of Vienna, Vienna, Austria

**Keywords:** peripheral nerve regeneration, nerve transfer, supercharged end-to-side, reversed end-to-side, innervation ratio

## Abstract

**Introduction:**

Peripheral nerve injuries often result in incomplete recovery, particularly after the occurrence of proximal lesions, owing to the extended reinnervation time as well as consequent reductions in the regeneration supportive factors and muscle recovery potential. In these cases, supercharged end-to-side (SETS) nerve transfers preserve the continuity of the original nerves while facilitating additional axonal support to mitigate muscle atrophy. This approach enhances functional recovery and has been demonstrated to be effective in both preclinical models and clinical settings. In this study, a novel SETS nerve transfer model is presented for the upper extremity of the rat to assess the impacts on muscle function, innervation ratio, and motor neuron regeneration as well as investigate the potential to enhance motor function recovery.

**Methods:**

The surgical interventions include transection and end-to-end repair of the musculocutaneous nerve (MCN) in Group A, transfer of the ulnar nerve (UN) to the side of the MCN in Group B, and a combination of both in Group C. The biceps muscle function was assessed 12 weeks post-surgery using electrical stimulation.

**Results:**

Muscle assessments revealed no significant differences in force between the experimental groups. UN-related muscle reinnervation was observed only in Group C after transfer to a regenerating nerve. Retrograde labeling demonstrated motor neuron regeneration of both the MCN and UN in a distal direction toward the muscle; however, tracer uptake of the UN motor neurons following intramuscular tracer application was detected only in Group C. In contrast, stained pseudounipolar cells in the dorsal root ganglia associated with the UN and MCN revealed afferent muscle innervations in Groups B and C.

**Discussion:**

This novel SETS nerve transfer model enables isolated electrophysiological as well as histological evaluations of all nerve sections to determine the muscle innervation ratio exactly. Our findings indicate that substantial functional efferent muscle innervation by the donor nerve is exclusively observed in a regenerating environment.

## 1 Introduction

In peripheral in-continuity nerve injuries or lesions on which primary end-to-end (ETE) repairs were performed, both sensory and motor functions often recover to certain degrees. However, proximal lesions particularly and frequently result in incomplete recovery with reduced functional outcomes owing to the greater reinnervation distance and time required for regeneration thereof (Fu and Gordon, 1995, 1997; Hoke, 2006). After denervation, the Schwann cells (SCs) undergo structural and functional modifications to create an environment supportive of nerve regeneration (Fu and Gordon, 1997; Glenn and Talbot, 2013; Wilcox et al., 2020). This condition is transient, and the expressions of the growth promoting factors diminish with prolonged denervation times (You et al., 1997; Jonsson et al., 2013). Moreover, when muscles are deprived of their neural connections owing to nerve injuries, a process of progressive atrophy and adipose tissue replacement occurs, which could become irreversible depending on the duration (Fu and Gordon, 1995; Weng et al., 2018).

In cases where the muscle functions remain unsatisfactory, distal ETE nerve transfers are considered the treatment option (Tung and Mackinnon, 2010). However, a major disadvantage of this technique is that the recipient nerve has to be cut. Therefore, the remaining residual function or ongoing regenerative capacity of the original nerve, even if minimal, is sacrificed. To preserve these potential capacities, the axons of the donor nerve can be introduced to the side of the affected recipient nerve to maintain its original continuity. This technique is referred to as the end-to-side (ETS) (Dellon et al., 2010), reversed end-to-side (RETS) (Isaacs et al., 2005; Kale et al., 2011; Isaacs, 2022; Pathiyil et al., 2023), or supercharged end-to-side (SETS) (Farber et al., 2013; von Guionneau et al., 2020) nerve transfer depending on literature. In the present work, we refer to it as SETS nerve transfer.

Neural regeneration following this procedure has been demonstrated in various preclinical studies in lower-limb rodent models (Isaacs et al., 2005; Fujiwara et al., 2007; Isaacs et al., 2008; Kale et al., 2011; Nadi et al., 2019). In principle, the objective is to achieve early reinnervation of the target muscles and SCs while preventing muscle atrophy, thereby improving motor recovery and regeneration of the recipient nerve. This so-called “babysitting” effect has been described in preclinical studies following the transfer of both sensory (Zuijdendorp et al., 2010; Li et al., 2014; Daniel et al., 2023) and motor (Farber et al., 2013; Zavala et al., 2023) nerves. Furthermore, the SETS transfer has also been described in clinical settings for restoration of intrinsic hand function following ulnar nerve impairment (Kale et al., 2011; Davidge et al., 2015; Baltzer et al., 2016; Koriem et al., 2020). In facial surgery, it has been applied for facial nerve palsy reconstruction using free functional muscle transfer to achieve dual innervation of the transplanted muscle (Biglioli et al., 2012; Sforza et al., 2015; Cardenas-Mejia et al., 2015).

In the present study, the effect of an SETS nerve coaptation was examined in a regenerative and native setting. Using a novel model for the upper extremity of the rat, we evaluated the impacts on muscle function and determined the innervation ratio between the nerves involved. Beyond its potential indication as a “babysitter,” the feasibility of augmenting and thus truly supercharging a muscle was investigated electrophysiologically and histologically.

## 2 Materials and methods

### 2.1 Animals

Twenty-nine male Sprague–Dawley rats aged 8–10 weeks were used in this study, where food and water were provided *ad libitum*. Intraoperative analgesia was achieved by subcutaneous administration of piritramide (0.3 mg/kg bodyweight). To maintain this effect, the animals received water supplemented with glucose and piritramide (30 mg of piritramide, 30 mL of 10% glucose, and 250 mL of water) postoperatively for 3 days and were monitored daily to assess recovery as well as detect signs of infection or weight loss. All experiments were approved by the local Institutional Committee for Animal Experimentation and the Austrian Federal Ministry of Education, Science and Research (BMBWF 2023-0.483.701).

### 2.2 Surgical procedures

All surgical procedures were performed under aseptic conditions by the same surgeon using an operating microscope. Anesthesia was induced by intraperitoneal injection of ketamine (100 mg/kg bodyweight) and xylazine (5 mg/kg bodyweight) and was maintained with 1.5% isoflurane following endotracheal intubation. All rats were placed in the supine position, and the abducted right upper limb was prepared for surgery. Subsequently, an incision was made over the major pectoral muscle lateral to the margin of the sternum, followed by muscle splitting and dissection of the brachial plexus.

**FIGURE 1:**
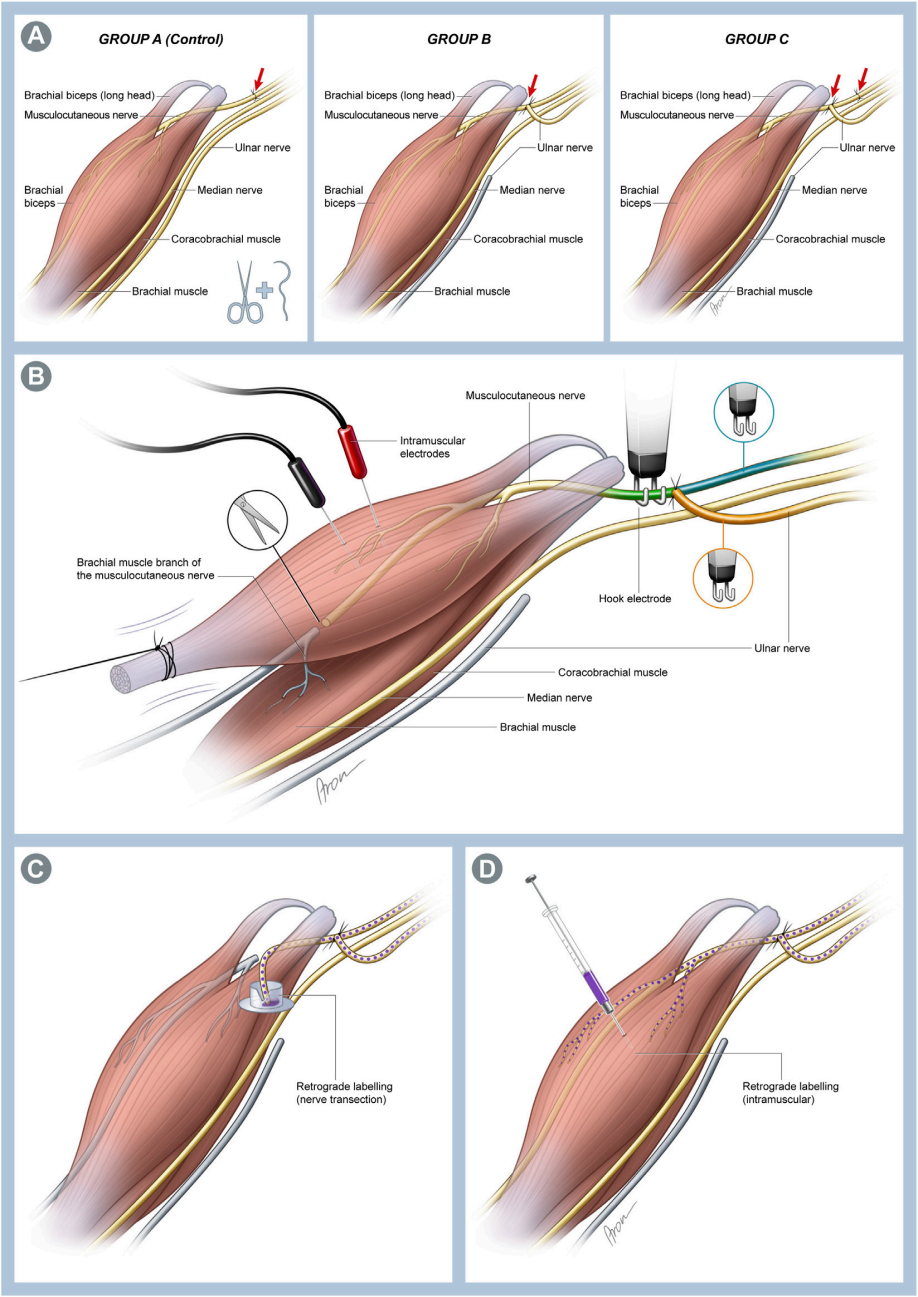
**(A)** Schematic illustration of the individual surgical groups. In Group A, which served as the control group, a transection and immediate end-to-end (ETE) repair of the musculocutaneous nerve (MCN) was performed. In Group B, the ulnar nerve (UN) was cut distally and transferred to the MCN in a supercharged end-to-side (SETS) fashion to enable investigation of this transfer to an intact nerve. Lastly, both interventions were combined in Group C to assess possible differences in a regenerating nerve following an SETS transfer. **(B)** Schematic of the muscle assessment setting. The biceps muscle (BM) was exposed over its full length up to insertion at the forearm. This was followed by identification and neurolysis of the MCN and UN. Then, the BM tendon was detached from the bone and attached to 3-0 silk suture before connection to a force transducer. The MCN was cut proximal to the brachialis muscle branches to avoid interfering contractions of the muscle. Two needle electrodes were placed in the muscle for recording EMG signals. Nerve stimulations were conducted using stainless-steel hook electrodes attached to the corresponding nerve sections to determine the innervation ratio. To achieve stimulation of all fibers innervating the muscle, the hook electrodes were placed on the MCN distal to the SETS coaptation site (MCNd, green) and proximal to the coaptation site for activation of the MCN (MCNp, blue) or UN (orange) fibers. **(C)** Schematic of the retrograde labeling used 12 weeks post-surgery. The MCN was transected immediately before its branches into the coracobrachial muscle (CBM), and the proximal stump was immersed in the cap of an Eppendorf tube filled with 4 µL of 2% Fast Blue (FB) to stain the neurons of the MCN and UN regenerating toward the muscle. **(D)** To identify the fibers of both nerves innervating the muscle, 5 µL of 2% FB was injected directly into both heads of the BM near the motor entry point using a 5-µL Hamilton microsyringe.

In Group A (n = 7), which served as the control group, the musculocutaneous nerve (MCN) was transected approximately 1–2 mm after emerging from the brachial plexus and immediately repaired in an ETE fashion with two 11-0 nylon single interrupted sutures. Group B (n = 9) animals were used to investigate the effects following an SETS transfer on an intact nerve. Here, the ulnar nerve (UN) was transected just before entering the cubital tunnel, and the proximal UN stump was transferred to the side of the MCN approximately 5 mm distal to its origin from the brachial plexus and 5 mm proximal to the coracobrachial muscle (CBM) via an epineural window using two 11-0 nylon single interrupted sutures. In Group C (n = 9), the MCN was transected and repaired as described in Group A, and the UN was transferred to the MCN as described in Group B 4 mm distal to the ETE repair site to provide a regenerative environment (Figure 1A). Thereafter, the pectoral muscle was repaired, and the incision was closed with 5-0 absorbable sutures in all groups. The animals recovered under a warming lamp and were observed for postoperative complications until they regained full consciousness. The remaining rats (n = 4) did not undergo any experimental surgery and were used for native nerve retrograde labeling.

### 2.3 Muscle force assessments

Twelve weeks post-surgery, the functional recovery of the biceps muscle (BM) was assessed by analyzing the forces generated during maximum evoked contraction (MEC) and maximum twitch (MT), along with registering the corresponding electromyography (EMG) signals. Force measurements were conducted on both the operated right side and native left side, with the latter serving as the internal control. Therefore, the rats were reanesthetized in the same manner described earlier. The MCN and UN were exposed as described above, followed by neurolysis of the nerves. Subsequently, the MCN was transected distal to its motor branches innervating the BM to prevent interfering contractions from the brachialis muscle. The BM was exposed over its full length up to the distal insertion on the forearm to allow access to the tendon. Great care was taken to preserve the supplying vessels.

For the force measurements, each rat was transferred onto a measurement table. The rat’s shoulder was positioned at approximately 90° abduction between two fixed supports, and the paw was secured with the limb extended to avoid arm movements. The tendon of the BM was then detached from its insertion and anchored through a clove hitch knot using a 3-0 silk suture. The knot was further secured using a small drop of superglue (Loctite Universal, Rocky Hill, CT, United States). The suture was then loosely attached to a force transducer (KD45 5N, ME Messsysteme, Henningsdorf, Germany). Two needle electrodes were placed in the muscle to obtain bipolar EMG recordings. Electrical stimulation was supplied using a custom-made stimulator (MiniVStim 18B, CTID, Center for Medical Physics and Biomedical Engineering, Medical University of Vienna, Austria) and delivered through bipolar stainless-steel hook electrodes attached to the nerve sections (Figure 1B). The current-controlled stimulator delivered supramaximal (2 mA, 400 μs) pseudomonophasic pulses with an exponential charge-balancing phase. To prevent drying of the nerve, a small cotton swab soaked in physiological saline solution was placed atop the nerve within the hook. Owing to the reduced electrical impedance, this moistened patch improved selectivity, thus avoiding the stimulation of adjacent nerve structures. All data were recorded using a PowerLab 16/35 device (ADInstruments, Sydney, Australia) at a sampling rate of 100 kS/s.

Approximately 15 min prior to the electrophysiological assessments, the isoflurane inhalation anesthesia was reduced to 1% to minimize any potential effects on muscle contractions. Prior to the actual measurements, the muscle length was adjusted using a custom-built automatic tensioning system to obtain a maximal twitch response during the supramaximal single-pulse stimulation. During the experiments, a series of three consecutive force measurements was obtained from different stimulation sites (Figure 1B). First, the hook electrode was placed on the MCN distal to the SETS coaptation site (MCNd) to stimulate all motor neurons reaching the BM. Thereafter, the UN and MCN proximal to the SETS coaptation site (MCNp) were stimulated separately to determine the innervation ratio between the nerves. On the native left side, the intact MCN was stimulated to obtain a baseline force for the healthy BM.

Each measurement evaluates the forces generated during MT and MEC. For the MT, three supramaximal pulses (A = 2 mA, PhW = 400 μs) were delivered at 1-s intervals between the pulses. For the MEC, three short bursts of supramaximal stimulation (A = 2 mA, PhW = 400 μs, F = 40 Hz, 330 ms duration) were administered at 30-s intervals between the bursts. The average of the three measurements was used as the final result. A minimum rest period of 3 min was maintained between consecutive sets of measurements to ensure complete muscle recovery. Upon completion of the muscle force assessments, the BMs from both the operated and non-operated contralateral side were harvested for weight analysis, and the animals were euthanized by intracardial application of 1 mL of pentobarbital.

### 2.4 Retrograde labeling

Retrograde labeling was conducted in all the groups as well as untreated animals, similar to previous works (Hayashi et al., 2007; Luft et al., 2021), to identify the origins of the motor axons in the spinal cord. To determine and distinguish the exact locations of the motor neuron populations of the MCN and UN in the spinal cord in untreated control animals (n = 4), both nerves were transected at the level of the upper arm approximately 10 mm distal to their origin from the plexus. Subsequently, both of the proximal nerve stumps were placed into the cap of an Eppendorf tube (Safe-Lock tube, Eppendorf, Germany) for 1 h, which was filled with either 4 µL of 10% Fluoro Ruby (FR; Invitrogen, Carlsbad, CA, United States) or 4 µL of 2% Fast Blue (FB; Polysciences, Warrington, PA, United States). To prevent tracer leakage and protect the nerves from being damaged by the rough tube edges, the reservoir was sealed with Vaseline (Fagron, Germany). To rule out any potential bias related to differences in the tracer characteristics, the tracers were alternated in half of the animals.

Twelve weeks after the primary surgery, the MCN was dissected using the same approach in Groups A (n = 2), B (n = 2), and C (n = 2). The MCN was first cut approximately 2 mm proximal to its entry into the CBM and distal to the SETS coaptation site, and the proximal stump was labeled with FB in the manner described above to stain both the MCN and UN fibers sprouting distally toward the muscles (Figure 1C). Additionally, in two other animals (Groups B and C, n = 1 each), the neurons directly innervating the BM were examined using a retrograde labeling injection technique, similar to the descriptions in previous works (Luft et al., 2021; Festin et al., 2024). Briefly, both heads of the BM were exposed, and a 5-µL Hamilton microsyringe was inserted to administer 5 µL of 2% FB into the muscle via multiple injections near the motor entry point (Figure 1D). The needle was retained inside the muscle for 1 min following each injection and retracted slowly to minimize potential leakage.

The animals were allowed to recover for 5–7 days following nerve labeling to ensure sufficient retrograde transport of the tracer. Then, the animals were deeply anesthetized using ketamine (200 mg/kg bodyweight) and xylazine (5 mg/kg bodyweight). Next, intracardial perfusion was performed via the left ventricle using 400 mL of 0.9% NaCl followed by 400 mL of 4% paraformaldehyde (PFA) solution. Subsequently, the spinal cord segments C4–Th2 were harvested in a single piece and immersed in 4% PFA solution for 24 h at 4°C and protected from light. The samples were rinsed with phosphate-buffered saline (PBS) for 24 h and dehydrated in glucose mixtures of increasing concentrations (10%, 25%, and 40% in PBS) for 24 h each. After the intramuscular tracer injections, the dorsal root ganglia (DRGs) C5, C6, C8, and Th1 were obtained from all animals and processed similarly, although they were stored in PFA for only approximately 8 h after perfusion owing to their sizes. Then, the samples were embedded in an optimal cutting temperature compound and stored at −80°C for at least 24 h. The spinal cord segments and DRGs were cut into longitudinal sections of 40 µm using a cryostat (Leica Microsystems, Germany) and transferred to slides. The motor neuron and pseudounipolar cell counts of each longitudinal spinal cord or ganglion section were obtained manually using a TissueFAXs slide scanner (TissueGnostics, Austria). The cells were counted when a nucleus and sufficient tracer uptake were clearly visible.

### 2.5 Data analysis and statistics

The recorded data were further processed using MATLAB (R2010a, The MathWorks Inc., MA, United States). For each force recording, the baseline force (i.e., average of 50 ms recording) was subtracted to obtain the active muscle force. For MT and MEC, the average values of the maximum active forces of three consecutive measurements were analyzed. For each EMG recording, the peak-to-peak (PTP) amplitude was determined for the first stimulation impulse. Given the challenges with consistent electrode placement, muscle size, and the resulting variability, the EMG outcomes were primarily utilized as qualitative evidence of muscle contractions. For the MT and MEC, the average PTP amplitudes of three consecutive measurements were analyzed.

To assess the extent of functional regeneration, a recovery index (RI) shown in Equation 1 was calculated for each animal. This was obtained by normalizing the peak forces generated by stimulation of the right experimental MCNd against the peak forces generated by stimulation of the left native MCN (reference). This normalization allowed comparison of the results across different animals while accounting for individual variations.

$$RI=\frac{\max\;\left(F_{active\_experimental}\right)}{\max\;\left(F_{active\_native}\right)}.$$ (1)

Additionally, the contribution ratios (CRs) shown in Equations 2, 3 were calculated to determine the individual contributions of the UN and MCNp with respect to the overall forces evoked by stimulation of the MCNd.

$${CR}_{UN}=\frac{\max\;\left(F_{active\_UN}\right)}{\max\;\left(F_{active\_MCNd}\right)}.$$ (2)

$${CR}_{{MCN}_{p}}=\frac{\max\;\left(F_{active\_MCNp}\right)}{\max\;\left(F_{active\_MCNd}\right).}$$ (3)

Statistical analysis was conducted using SPSS (version 27; IBM, United States). The group results were displayed as means ± standard deviations. The muscle weight and MEC values of all groups were tested for normality of distribution (Shapiro–Wilk test). As the results were normally distributed, ANOVA was performed to compare the RIs of all groups, and the t-test was used to compare the CRs of different groups (unpaired) or RIs within groups (paired). The level of significance was set to α = 0.05. As the MEC was defined as our primary parameter of muscle force outcome, statistical tests were only conducted for this outcome. Histological evaluations were performed as a qualitative verification and were represented descriptively.

## 3 Results

### 3.1 Muscle weight

**FIGURE 2:**
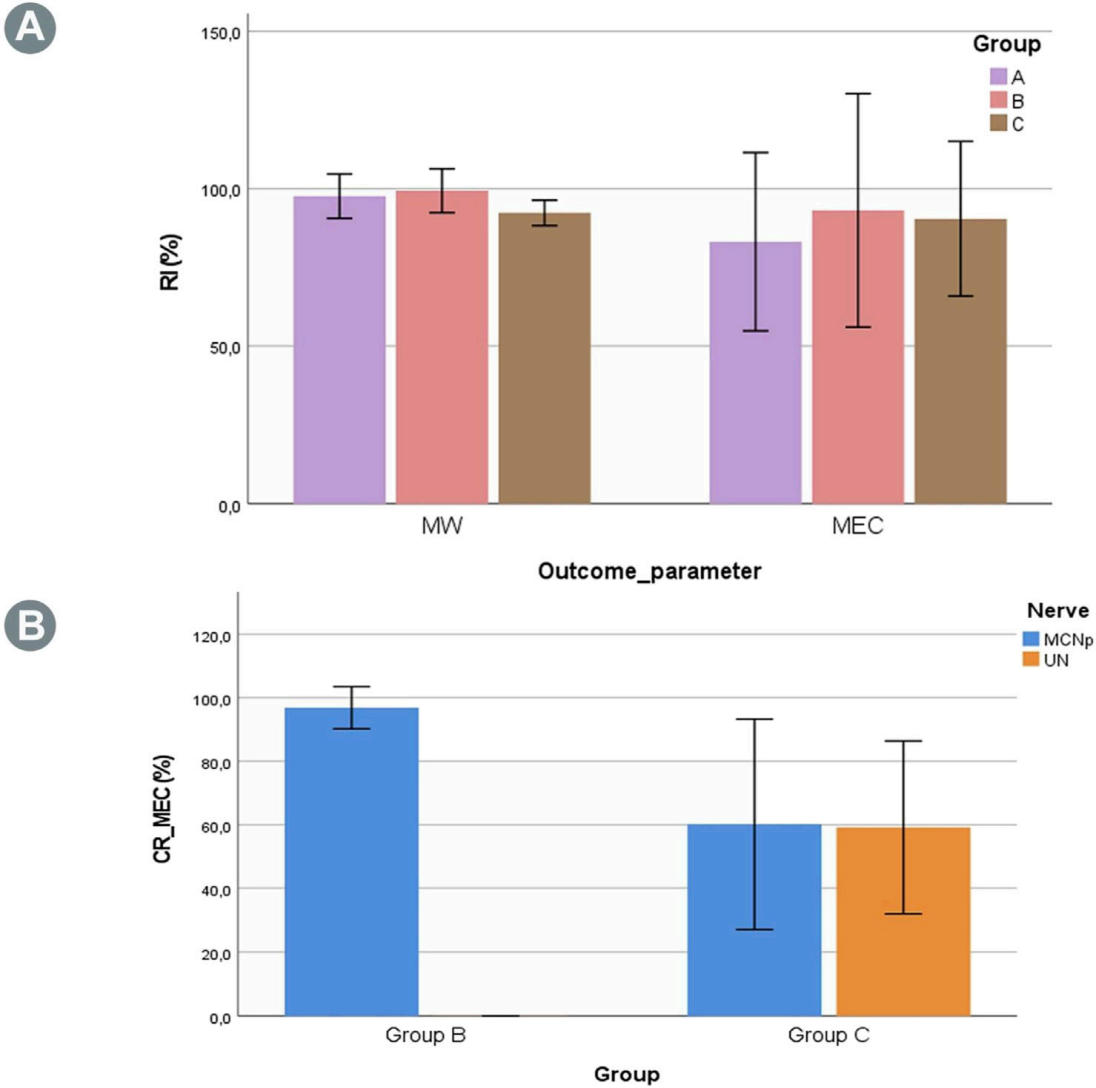
**(A)** Recovery indexes (RIs) for the muscle weight (MW) and maximum evoked contraction (MEC) of the BM in the three groups. No statistically significant differences were found between the groups. **(B)** Contribution ratio (CR) of the MCNd elicited MECs generated by the UN or MCNp. In Group B, no involvement of the UN was observed for muscle innervation, while the contribution in Group C was almost evenly distributed between the UN and MCNp, albeit with a marked variability.

The dry BM weight of the experimental right side was compared to the native left side as the RI (Equation 1). Table 1 shows this index as well as the absolute muscle and animal weights of the various groups. The outcomes showed no statistically significant differences for RIs between the groups (*p* = 0.16) (Figure 2A).

**TABLE 1:** Summary of animals and BM weights.

	Group A		Group B		Group C	
Rat weight (g)	713.6 ± 40.1		651.2 ± 76.1		645.8 ± 47.6	
Muscle weight (mg)	Left	Right	Left	Right	Left	Right
	485.6 ± 25.8	473.6 ± 50.6	471.3 ± 84.8	467.0 ± 78.9	475.7 ± 52.6	439.5 ± 55.6
RI (%)	97.6 ± 7.0		99.4 ± 7.0		92.3 ± 4.1	

### 3.2 Muscle force assessments

The EMG PTP amplitudes as well as MEC and MT assessments after stimulation of the MCNd, MCNp, and UN were also expressed in terms of the RI, as described above. Additionally, these outcome parameters were expressed in terms of the CR following selective stimulation of the UN or MCNp to represent their corresponding contributions compared to the MCNd outcomes. A detailed summary of all outcomes is provided in Table 2.

**TABLE 2:** Summary of the electrophysiological BM assessment outcomes.

	Group A			Group B								Group C						
	Left	Right	RI%	Left	Right			RI%	CR_MCNp_%	CR_UN_%	Left		Right			RI%	CR_MCNp_%	CR_UN_%
	MCN	MCN		MCN	MCNd	MCNp	UN				MCN		MCNd	MCNp	UN			
MEC (N)	1.228 ± 0.195	0.985 ± 0.229	83.2 ± 28.3	1.179 ± 0.286	1.084 ± 0.403	1.053 ± 0.390	0	93.1 ± 37.1	96.9 ± 6.6	0	1.079 ± 0.211		0.958 ± 0.264	0.549 ± 0.311	0.577 ± 0.308	90.4 ± 24.6	60.2 ± 33.1	59.2 ± 27.2
MT (N)	0.495 ± 0.066	0.375 ± 0.123	78.0 ± 29.9	0.483 ± 0.088	0.460 ± 0.188	0.446 ± 0.182	0	94.5 ± 35.1	96.8 ± 2.6	0	0.481 ± 0.088		0.358 ± 0.143	0.203 ± 0.152	0.203 ± 0.121	73.6 ± 23.2	59.4 ± 35.4	55.4 ± 27.0
EMG PTP (mV)	22.3 ± 10.5	11.2 ± 7.5	50.2 ± 26.2	12.3 ± 8.9	14.1 ± 9.0	13.5 ± 9.0	0	139.4 ± 81.8	92.6 ± 12.7	0	13.1 ± 9.5		17.4 ± 12.0	12.7 ± 14.0	7.7 ± 4.5	342.9 ± 613.9	65.6 ± 41.3	50.4 ± 35.7

The muscle force assessments demonstrated reproducible muscle responses of the right BMs in all animals of Group A (n = 5) for MCN stimulation, with minor differences compared to the non-operated side (Table 2). In Group B (n = 6) EMG, MEC and MT were recorded after stimulating the MCNd, thus activating all the fibers of the MCN and UN innervating the BM. Analysis of the MEC measurements revealed slightly reduced outcomes in comparison to the unoperated side (Table 2). For the MEC, only minimal and statistically insignificant differences were observed compared to MCNp stimulation, which selectively targeted the MCN fibers (*p* = 0.34) with an MCNp CR of 96.9 ± 6.9. However, stimulation of the UN did not elicit any muscle responses or EMG signals (Figure 2B). In contrast, BM contractions were detected at all stimulation sites (MCNd, MCNp, and UN) in Group C (n = 6), with greater MECs when comparing MCNd to MCNp stimulation. This difference was statistically significant (*p* = 0.04). The UN-associated MEC was similar to the MCNp-related MEC (*p* = 0.90). For the MEC CR, 59.2% ± 27.2% were related to the UN and 60.2% ± 33.1% were related to the MCNp, representing minimal and statistically insignificant higher contribution of the MCNp (*p* = 0.97) (Figure 2B).

Evaluation of the MEC RIs across all three groups following stimulation of the MCN or MCNd revealed statistically insignificant differences (*p* = 0.86) (Figure 2A). Stimulation of the UN in Group B showed no muscle responses, resulting in a statistically significant lower CR compared to Group C (*p* < 0.01).

### 3.3 Retrograde labeling

#### 3.3.1 Native motor neuron population

**FIGURE 3:**
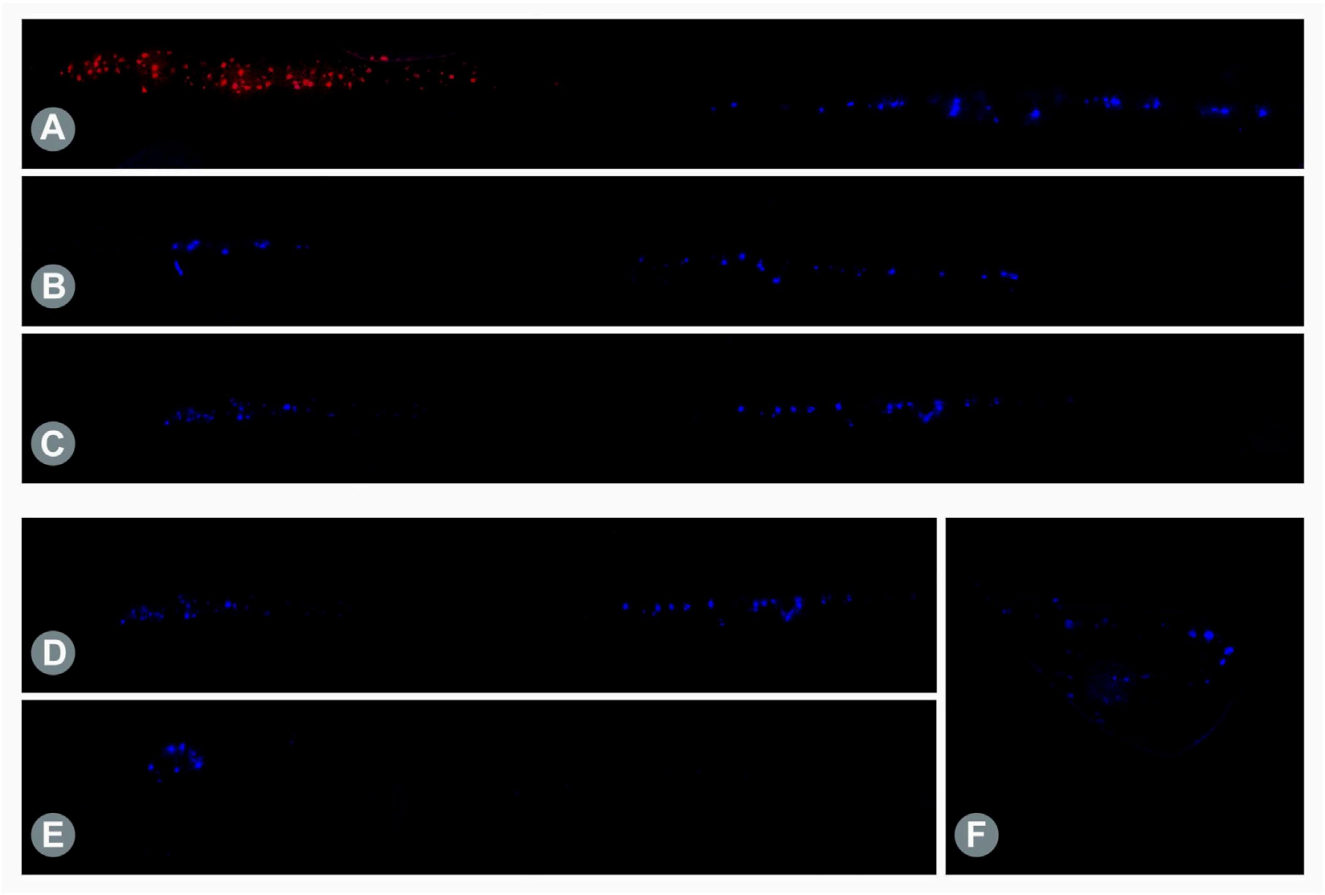
**(A)** Retrogradely labeled motor neurons in the longitudinal spinal cord sections. Staining of native MCN (red) and UN (blue) cells using Fluoro Ruby (FR) and FB showed a clear segmental separation in the spinal cord, with the MCN-associated motor neurons predominantly present in C5/C6 and UN-related neurons present in C8/Th1. **(B)** Twelve weeks post-surgical retrograde labeling of the proximal nerve stump after transection of the MCN distal to the SETS coaptation site revealed motor neuron regeneration of both the MCN (left cell pool) and UN (right cell pool) in a distal direction toward the muscle after SETS transfer to an intact nerve in Group B and **(C)** to a regenerating nerve in Group C. **(D)** Retrogradely labeled motor neurons in the longitudinal spinal cord sections after intramuscular tracer application. Following intramuscular injection of 5 µL of 2% FB into the BM 12 weeks after SETS transfer, tracer uptake was identified in the motor neuron pool of the MCN (left cell pool) and UN (right cell pool) in Group C. **(E)** In accordance with the electrophysiological results, only MCN-associated motor neurons were stained in the spinal cord and no stained neurons were noted in the UN segments in Group B. **(F)** However, despite the missing efferent innervation by the UN, labeled pseudounipolar neurons were observed in the DRGs C8/Th1.

Retrograde labeling of the UN and MCN using two different tracers (FB and FR) in the untreated control animals showed a clear separation between the motor neuron populations in the spinal cord segments (Figure 3A), with a total of 162 ± 17 neurons located predominantly at the C8/Th1 level (UN) and 158 ± 7 neurons located at the C5/C6 level (MCN) (Table 3). This segmental separation allowed categorization of the two nerves based on the spinal cord segment level in the following analyses.

**TABLE 3:** Summary of the motor neuron or DRG cell counts following retrograde labeling after distal nerve transection (nt) of the MCN or intramuscular (im) tracer application.

Nerve	Native	Group A	Group B			Group C		
			nt	im	im-DRG	nt	im	im-DRG
MCN	158 ± 7	139 ± 6	179 ± 4	56	153	146 ± 30	51	198
UN	162 ± 17	-	82 ± 21	0	127	101 ± 7	46	221

#### 3.3.2 Motor fiber regeneration following SETS transfer

After transection and retrograde staining of the proximal MCN (Group A) or MCNd (Groups B and C) stump, labeled motor neurons were identified in the spinal cord segments associated with the MCN in all groups, with the highest count being observed in Group B and followed by Group C. In Groups B and C, additional motor neuron cell bodies were detected in the spinal cord levels corresponding to the UN after SETS nerve coaptation (Figures 3B, C), with a higher number of cells found in Group C (Table 3). The detailed cell count numbers are displayed in Table 3.

#### 3.3.3 Intramuscular application

Retrogradely stained neurons following intramuscular tracer injection into the BM were found in the spinal cord segments and DRGs of both the MCN and UN in Group C (Figure 3D). In this regenerating SETS nerve transfer model, the quantifications revealed 46 (UN) and 51 (MCN) cells in the spinal cord as well as 166 (UN) and 149 (MCN) cells in the DRGs (Table 3). However, in Group B, 56 motor neurons were found to be associated only with the MCN in the spinal cord, but none corresponded to the UN (Figure 3E). Interestingly, 115 and 96 stained pseudounipolar cells were identified in the DRGs of the UN and MCN, respectively (Figure 3F).

## 4 Discussion

Nerve transfer techniques that maintain the integrity of the donor or recipient nerve are rapidly garnering interest as they enable preservation of the original innervation or reduction of donor-site morbidity. This concept was reintroduced and first popularized by Viterbo et al. (1992), who transferred the distal end of the recipient nerve to the side of an intact donor, and later applied in a reversed fashion by Isaacs et al. (2005) to preserve the continuity of the recipient nerve. It is worth noting that there are discrepancies in literature concerning the nomenclature of the SETS transfer. Some authors, including the original describers (Isaacs et al., 2005; Kale et al., 2011; Pathiyil et al., 2023), refer to the procedure as RETS to emphasize the role reversal compared to the technique described in Viterbo’s study. Dellon et al. (2010) define it as ETS according to the general nomenclature for nerve transfers based on the principle of “*coming from and going to.*” To simplify identification within literature while adhering to Dellon’s systematic nomenclature, we adopted the previously proposed term SETS (Farber et al., 2013; von Guionneau et al., 2020; Zavala et al., 2023; Daniel et al., 2023) as a reasonable compromise.

In this study, we introduced a novel SETS model for the upper limb of the rat to investigate this transfer in both an intact and a regenerating environment to evaluate it in electrophysiological terms compared to a control group. In contrast to existing SETS rodent models of the lower limb, which aimed for selectivity in proximal stimulation through nerve transection (Isaacs et al., 2005; Isaacs et al., 2008), our model allows direct isolated stimulation and individual assessment of all nerve sections within an animal to accurately determine the innervation ratio of the target muscle. Furthermore, the contralateral extremity is preserved for internal control, unlike models necessitating nerve transfer from the opposite sides to facilitate isolated nerve stimulation (Fujiwara et al., 2007).

The choice of an epineural or a perineural window during the lateral coaptation is still not fully clear. Reliable innervation results have been reported in literature following opening of the epineurium (Isaacs et al., 2005; Fujiwara et al., 2007; Nadi et al., 2019) as well as the perineurium (Kale et al., 2011; Farber et al., 2013; Daniel et al., 2023). In a rodent study conducted by Chu et al. (2023), histological analysis revealed enhanced axonal regeneration following a SETS transfer via a perineural window. However, in our opinion, the feasibility of precisely distinguishing connective tissue disruption in this model remains debatable, given the small caliber as well as the fact that the connective tissue layers partially merge into one another and are not delineated clearly, especially in monofascicular nerves. Thus, an epineural window may potentially coinduce a perineural injury to a certain degree, and a perineural opening may similarly affect the nerve fibers. The primary objective of our model was to examine possible augmentation in addition to and with preservation of the recipient nerve innervation to assess differences between fully intact and freshly regenerating nerve fibers. Hence, we opted to perform an epineural window to minimize damage to the recipient nerve and distortion of the results thereof; this avoids damage to the nerve while simultaneously promoting regeneration (Liu et al., 1999).

In previous preclinical studies, the functional outcome measured as muscle force after an SETS transfer in peripheral nerve reconstruction was superior to that of the corresponding control group (Fujiwara et al., 2007; Farber et al., 2013; Isaacs et al., 2019). Comparing Groups A and C in terms of motor recovery outcomes (i.e., muscle strength and muscle weight), we observed no significant differences in this study and only a slight increase in MEC in Group C over Group A, similar to the findings of Isaacs et al. (2008). One possible reason for this may be that no major difference was detected within control Group A between the experimental and healthy unaffected sides, so no relevant improvement was further detected in Group C. Additionally, the “babysitting effect” may not be substantial given the short regeneration distance, in contrast to the lower extremity (Fujiwara et al., 2007) or interposition graft (Farber et al., 2013; Isaacs et al., 2019) models, where the recipient nerve regeneration is impeded by overcoming two coaptation sites. This limitation is presumably further confounded by the excellent and rapid nerve regeneration abilities of rats. However, outcomes such as MT force and muscle weight were reduced in animals following an additional SETS transfer compared to those receiving only an ETE repair. This may be attributed to the aforementioned hypothesis that the donor nerves “*compete with rather than augment*” (Isaacs, 2022), thereby hindering native nerve regeneration, especially given the proximity. These findings suggest that an SETS transfer, when considered in its indication as a babysitter, is only recommended for proximal lesions with extended regeneration times.

Furthermore, it is notable that distal stimulations of all fibers in the animals of Group C were inferior to the summation of stimulating the individual nerves. This observation is consistent with the findings of Isaacs et al. (2008), indicating that individual muscle fibers may be innervated by both nerves and may require further histological investigations. In Group B, the total muscle force on the experimental side reduced after an additional SETS transfer to the intact nerve compared to the contralateral unoperated side. This could be due to MCN impairment by the surgical scar or physical interference caused by the histologically proven ingrown fibers of the UN.

To determine the origin of the motor fibers distal to the coaptation site, retrograde labeling was used after mapping the native motor neuron pools of the respective nerves. Since the electrophysiological assessments of Group B revealed no activity following UN stimulation, the examination of these fibers was of particular interest. Our histological findings confirmed motor fiber presence for both the donor and recipient nerves in a distal direction toward the muscle after SETS transfer in all groups. Donor nerve regeneration via SETS coaptation after transfer to a regenerating nerve has been histologically validated previously (Fujiwara et al., 2007; Nadi et al., 2019; Chu et al., 2023) but not in an intact nerve. Although Isaacs et al. (2005) conducted histological examinations of the nerve cross-sections in the region of the SETS coaptation site, they acknowledged that this approach could not provide definite proof of origin of the donor or recipient axons. Following motor neuron identification after distal nerve transection, we aimed to further investigate potential end-organ reinnervation through an intramuscular injection of the tracer directly into the BM. Consistent with the muscle assessment results, stained motor neurons were found in the spinal cord levels corresponding to the MCN and UN in Group C, whereas no UN-associated motor neurons were observed in Group B, leaving the exact fates of these fibers undetermined.

Following intramuscular tracer application, the DRGs C5/C6 (MCN) and C8/Th1 (UN) were additionally harvested from the animals to assess the regenerative characteristics of the afferent axons. In Group C, labeled cells were identified in all four DRGs, similar to the motor neurons in the spinal cord. Notably, in Group B, despite no electrophysiological and histological detection of efferent muscle innervation by the UN, stained perikarya of pseudounipolar cells were observed in the DRGs C8 and Th1. These findings indicate that following SETS transfer to an intact nerve, only the afferent proprioceptive fibers may reach and innervate a motoric target organ. However, the small sample size in this work limits the validity of this conclusion.

The objective of this study was to not only evaluate the clinical indication of an SETS transfer as a babysitter after initial nerve reconstruction but also explore its potential for augmenting muscle functions at later stages in the presence of reduced yet existing motor recovery, such as in cases after neurolysis as a primary approach or delayed nerve reconstruction. In the first step, we assessed the potential differences in nerve regeneration, innervation ratio, and impacts on muscle functions between freshly regenerating and non-injured intact nerves. Immediately after SETS transfer following a nerve lesion, the donor nerve regeneration presumably benefits from the proregenerative environment created by the SCs of the damaged recipient nerve. However, these promoting factors decrease over time (You et al., 1997; Jonsson et al., 2013) and are no longer present substantially approximately 6 months post-injury (Sulaiman and Gordon, 2013). Thus, we initially sought to examine whether regeneration occurs within the recipient and assessed the potential involvement of innervation following transfer to an intact nerve, where a regeneration-promoting environment is absent. In the case of incomplete regeneration, collateral sprouting enables the axons to innervate up to five times the number of muscle fibers (Fu and Gordon, 1995), thus enabling innervation of the majority of muscle fibers through enlarging the motor units even in cases of substantial loss of neuronal capacity. Hence, it was of interest to determine if already innervated fibers could be taken over or additionally innervated by a second nerve simultaneously, thereby increasing the neural input in a supercharged manner. We found evidence of donor nerve participation in a regenerative setting but observed neither functional nor histological involvement in motor innervation of the muscle in an intact nerve. Given the observed discrepancies, a possible next step could be to explore this procedure in an incomplete regeneration model, such as that described previously (Kalliainen et al., 2002; Isaacs et al., 2013), with a delayed additional SETS transfer to fully reflect the potential clinical applications. Alternatively, a proximal partial crush of the intact nerve could be conducted to determine whether this improves donor nerve regeneration and enables solid muscle innervation.

## 5 Conclusion

Nerve regeneration via SETS nerve coaptation occurs in both regenerating and intact environments. However, substantial efferent muscle innervation was observed exclusively in the regenerative setting. The proposed babysitting effect, which is hypothesized to enhance donor nerve regeneration and muscle recovery, could not be demonstrated in our study owing to the short regeneration distance. The observation of absent motor innervation involvement following transfer to an intact nerve necessitates further research to critically evaluate the indications for augmenting muscle functions at a later point in time following diminished motor recovery.

## Data Availability

The original contributions presented in this study are included in the article/Supplementary Material, and any further inquiries may be directed to the corresponding author.

## References

[B1] Baltzer H. Woo A. Oh C. Moran S. L. (2016). Comparison of ulnar intrinsic function following supercharge end-to-side anterior interosseous-to-ulnar motor nerve transfer: a matched cohort study of proximal ulnar nerve injury patients. Plast. Reconstr. Surg. 138 (6), 1264–1272. 10.1097/PRS.0000000000002747 27879594

[B2] Biglioli F. Colombo V. Tarabbia F. Pedrazzoli M. Battista V. Giovanditto F. (2012). Double innervation in free-flap surgery for long-standing facial paralysis. J. Plast. Reconstr. Aesthet. Surg. 65 (10), 1343–1349. 10.1016/j.bjps.2012.04.030 22728067

[B3] Cardenas-Mejia A. Covarrubias-Ramirez J. V. Bello-Margolis A. Rozen S. (2015). Double innervated free functional muscle transfer for facial reanimation. J. Plast. Surg. Hand Surg. 49 (3), 183–188. 10.3109/2000656X.2014.988218 25469588

[B4] Chu T. H. Alzahrani S. McConnachie A. Lasaleta N. Kalifa A. Pathiyil R. (2023). Perineurial window is critical for experimental reverse end-to-side nerve transfer. Neurosurgery 93 (4), 952–960. 10.1227/neu.0000000000002481 37018413

[B5] Daniel B. W. Giesen T. Lu J. C. Chang T. N. Zavala A. Chuang D. C. (2023). Supercharge end-to-side sensory transfer to A long nerve graft to enhance motor regeneration in A brachial plexus model-an experimental rat study. J. Reconstr. Microsurg 39 (6), 435–443. 10.1055/s-0042-1758186 36451622

[B6] Davidge K. M. Yee A. Moore A. M. Mackinnon S. E. (2015). The supercharge end-to-side anterior interosseous-to-ulnar motor nerve transfer for restoring intrinsic function: clinical experience. Plast. Reconstr. Surg. 136 (3), 344e–352e. 10.1097/PRS.0000000000001514 26313839

[B7] Dellon A. L. Ferreira M. C. Williams E. H. Rosson G. D. (2010). Which end is up? Terminology for terminolateral (end-to-side) nerve repair: a review. J. Reconstr. Microsurg 26 (5), 295–301. 10.1055/s-0030-1248240 20143303

[B8] Farber S. J. Glaus S. W. Moore A. M. Hunter D. A. Mackinnon S. E. Johnson P. J. (2013). Supercharge nerve transfer to enhance motor recovery: a laboratory study. J. Hand Surg. Am. 38 (3), 466–477. 10.1016/j.jhsa.2012.12.020 23391355 PMC3583195

[B9] Festin C. Ortmayr J. Maierhofer U. Tereshenko V. Blumer R. Schmoll M. (2024). Creation of a biological sensorimotor interface for bionic reconstruction. Nat. Commun. 15 (1), 5337. 10.1038/s41467-024-49580-8 38914540 PMC11196281

[B10] Fu S. Y. Gordon T. (1995). Contributing factors to poor functional recovery after delayed nerve repair: prolonged denervation. J. Neurosci. 15 (5 Pt 2), 3886–3895. 10.1523/JNEUROSCI.15-05-03886.1995 7751953 PMC6578254

[B11] Fu S. Y. Gordon T. (1997). The cellular and molecular basis of peripheral nerve regeneration. Mol. Neurobiol. 14 (1-2), 67–116. 10.1007/BF02740621 9170101

[B12] Fujiwara T. Matsuda K. Kubo T. Tomita K. Hattori R. Masuoka T. (2007). Axonal supercharging technique using reverse end-to-side neurorrhaphy in peripheral nerve repair: an experimental study in the rat model. J. Neurosurg. 107 (4), 821–829. 10.3171/JNS-07/10/0821 17937230

[B13] Glenn T. D. Talbot W. S. (2013). Signals regulating myelination in peripheral nerves and the Schwann cell response to injury. Curr. Opin. Neurobiol. 23 (6), 1041–1048. 10.1016/j.conb.2013.06.010 23896313 PMC3830599

[B14] Hayashi A. Moradzadeh A. Hunter D. A. Kawamura D. H. Puppala V. K. Tung T. H. (2007). Retrograde labeling in peripheral nerve research: it is not all black and white. J. Reconstr. Microsurg 23 (7), 381–389. 10.1055/s-2007-992344 17979067

[B15] Hoke A. (2006). Mechanisms of Disease: what factors limit the success of peripheral nerve regeneration in humans? Nat. Clin. Pract. Neurol. 2 (8), 448–454. 10.1038/ncpneuro0262 16932603

[B16] Isaacs J. (2022). Reverse end-to-side (supercharging) nerve transfer: conceptualization, validation, and translation. Hand (N Y) 17 (6), 1017–1023. 10.1177/1558944720988076 33530769 PMC9608296

[B17] Isaacs J. Allen D. Chen L. E. Nunley J. (2005). Reverse end-to-side neurotization. J. Reconstr. Microsurg 21 (1), 43–50. 10.1055/s-2005-862780 15672319

[B18] Isaacs J. Mallu S. Wo Y. Shah S. (2013). A rodent model of partial muscle re-innervation. J. Neurosci. Methods 219 (1), 183–187. 10.1016/j.jneumeth.2013.07.019 23928151

[B19] Isaacs J. Patel G. Mallu S. Ugwu-Oju O. Desai A. Borschel G. (2019). Effect of reverse end-to-side (supercharging) neurotization in long processed acellular nerve allograft in a rat model. J. Hand Surg. Am. 44 (5), 419 e1–e419 e10. 10.1016/j.jhsa.2018.07.008 30172450

[B20] Isaacs J. E. Cheatham S. Gagnon E. B. Razavi A. McDowell C. L. (2008). Reverse end-to-side neurotization in a regenerating nerve. J. Reconstr. Microsurg 24 (7), 489–496. 10.1055/s-0028-1088230 18803150

[B21] Jonsson S. Wiberg R. McGrath A. M. Novikov L. N. Wiberg M. Novikova L. N. (2013). Effect of delayed peripheral nerve repair on nerve regeneration, Schwann cell function and target muscle recovery. PLoS One 8 (2), e56484. 10.1371/journal.pone.0056484 23409189 PMC3567071

[B22] Kale S. S. Glaus S. W. Yee A. Nicoson M. C. Hunter D. A. Mackinnon S. E. (2011). Reverse end-to-side nerve transfer: from animal model to clinical use. J. Hand Surg. Am. 36 (10), 1631–1639 e2. 10.1016/j.jhsa.2011.06.029 21872405

[B23] Kalliainen L. K. Jejurikar S. S. Liang L. W. Urbanchek M. G. Kuzon W. M. Jr. (2002). A specific force deficit exists in skeletal muscle after partial denervation. Muscle Nerve 25 (1), 31–38. 10.1002/mus.1216 11754182

[B24] Koriem E. El-Mahy M. M. Atiyya A. N. Diab R. A. (2020). Comparison between supercharged ulnar nerve repair by anterior interosseous nerve transfer and isolated ulnar nerve repair in proximal ulnar nerve injuries. J. Hand Surg. Am. 45 (2), 104–110. 10.1016/j.jhsa.2019.11.005 31866151

[B25] Li Q. Zhang P. Yin X. Han N. Kou Y. Jiang B. (2014). Early sensory protection in reverse end-to-side neurorrhaphy to improve the functional recovery of chronically denervated muscle in rat: a pilot study. J. Neurosurg. 121 (2), 415–422. 10.3171/2014.4.JNS131723 24878291

[B26] Liu K. Chen L. E. Seaber A. V. Goldner R. V. Urbaniak J. R. (1999). Motor functional and morphological findings following end-to-side neurorrhaphy in the rat model. J. Orthop. Res. 17 (2), 293–300. 10.1002/jor.1100170220 10221848

[B27] Luft M. Klepetko J. Muceli S. Ibanez J. Tereshenko V. Festin C. (2021). Proof of concept for multiple nerve transfers to a single target muscle. Elife 10, e71312. 10.7554/eLife.71312 34596042 PMC8530510

[B28] Nadi M. Ramachandran S. Islam A. Forden J. Guo G. F. Midha R. (2019). Testing the effectiveness and the contribution of experimental supercharge (reversed) end-to-side nerve transfer. J. Neurosurg. 130 (3), 702–711. 10.3171/2017.12.JNS171570 29775143

[B29] Pathiyil R. K. Alzahrani S. Midha R. (2023). Reverse end-to-side transfer to ulnar motor nerve: evidence from preclinical and clinical studies. Neurosurgery 92 (4), 667–679. 10.1227/neu.0000000000002325 36757319

[B30] Sforza C. Frigerio A. Mapelli A. Tarabbia F. Annoni I. Colombo V. (2015). Double-powered free gracilis muscle transfer for smile reanimation: a longitudinal optoelectronic study. J. Plast. Reconstr. Aesthet. Surg. 68 (7), 930–939. 10.1016/j.bjps.2015.03.029 26026222

[B31] Sulaiman W. Gordon T. (2013). Neurobiology of peripheral nerve injury, regeneration, and functional recovery: from bench top research to bedside application. Ochsner J. 13 (1), 100–108. Available at: https://www.ncbi.nlm.nih.gov/pubmed/23531634. 23531634 PMC3603172

[B32] Tung T. H. Mackinnon S. E. (2010). Nerve transfers: indications, techniques, and outcomes. J. Hand Surg. Am. 35 (2), 332–341. 10.1016/j.jhsa.2009.12.002 20141906

[B33] Viterbo F. Trindade J. C. Hoshino K. Mazzoni Neto A. (1992). Latero-terminal neurorrhaphy without removal of the epineural sheath. Experimental study in rats. Rev. Paul. Med. 110 (6), 267–275. Available at: https://www.ncbi.nlm.nih.gov/pubmed/1341024. 1341024

[B34] von Guionneau N. Sarhane K. A. Brandacher G. Hettiaratchy S. Belzberg A. J. Tuffaha S. (2020). Mechanisms and outcomes of the supercharged end-to-side nerve transfer: a review of preclinical and clinical studies. J. Neurosurg. 134 (5), 1590–1598. 10.3171/2020.3.JNS191429 32470926

[B35] Weng J. Zhang P. Yin X. Jiang B. (2018). The whole transcriptome involved in denervated muscle atrophy following peripheral nerve injury. Front. Mol. Neurosci. 11, 69. 10.3389/fnmol.2018.00069 29563865 PMC5845901

[B36] Wilcox M. B. Laranjeira S. G. Eriksson T. M. Jessen K. R. Mirsky R. Quick T. J. (2020). Characterising cellular and molecular features of human peripheral nerve degeneration. Acta Neuropathol. Commun. 8 (1), 51. 10.1186/s40478-020-00921-w 32303273 PMC7164159

[B37] You S. Petrov T. Chung P. H. Gordon T. (1997). The expression of the low affinity nerve growth factor receptor in long-term denervated Schwann cells. Glia 20 (2), 87–100. 10.1002/(sici)1098-1136(199706)20:2<87::aid-glia1>3.0.co;2-1 9179594

[B38] Zavala A. Lu J. C. Chang T. N. Daniel B. W. Chuang D. C. (2023). Supercharge end-to-side motor transfer to a long nerve graft to enhance motor regeneration: an experimental rat study. Plast. Reconstr. Surg. 152 (1), 85e–95e. 10.1097/PRS.0000000000010114 36728802

[B39] Zuijdendorp H. M. Tra W. M. van Neck J. W. Mollis L. Coert J. H. (2010). Delay of denervation atrophy by sensory protection in an end-to-side neurorrhaphy model: a pilot study. J. Plast. Reconstr. Aesthet. Surg. 63 (12), 1949–1952. 10.1016/j.bjps.2010.01.018 20303842

