# Supplementary material for: Axonal regeneration and innervation ratio following supercharged end-to-side nerve transfer
Source: Front Cell Dev Biol. 2025 Feb 12;13:1513321. doi: 10.3389/fcell.2025.1513321 (PMC11860980; doi:10.3389/fcell.2025.1513321)
Supplement: Supplementary file 1 [file Table1.docx]

**Suppl. Tables**

**Table 1a – Rat weight (g)**

| **Animal** | **Group A** | **Group B** | **Group C** |
| --- | --- | --- | --- |
| 1 | 668 | 660 | 598 |
| 2 | 678 | 573 | 639 |
| 3 | 727 | 665 | 601 |
| 4 | 765 | 787 | 697 |
| 5 | 730 | 590 | 630 |
| 6 | - | 632 | 710 |

**Table 1b – Muscle weight (mg)**

| **Animal** | **Group A** | | | **Group B** | | | **Group C** | | |
| --- | --- | --- | --- | --- | --- | --- | --- | --- | --- |
|  | **Left** | **Right** | **RI (%)** | **Left** | **Right** | **RI (%)** | **Left** | **Right** | **RI (%)** |
| 1 | 468 | 441 | 94.2 | 388 | 374 | 96.4 | 411 | 368 | 89.5 |
| 2 | 464 | 442 | 95.3 | 429 | 414 | 96.5 | 426 | 397 | 93.2 |
| 3 | 508 | 559 | 110.0 | 578 | 551 | 95.3 | 520 | 518 | 99.6 |
| 4 | 519 | 481 | 92.7 | 580 | 563 | 97.1 | 544 | 487 | 89.5 |
| 5 | 469 | 445 | 94.9 | 423 | 412 | 97.4 | 460 | 429 | 93.3 |
| 6 | - | - | - | 430 | 488 | 113.5 | 493 | 438 | 88.8 |

**Table 2a – MEC (N)**

| **Animal** | **Group A** | | | **Group B** | | | | | | | **Group C** | | | | | | |
| --- | --- | --- | --- | --- | --- | --- | --- | --- | --- | --- | --- | --- | --- | --- | --- | --- | --- |
|  | **MEC** | | | **MEC** | | | | | | | **MEC** | | | | | | |
|  | Left | Right | RI (%) | Left | Right | | | RI (%) | CR_MCNp_ (%) | CR_UN_ (%) | Left | Right | | | RI (%) | CR_MCNp_ (%) | CR_UN_ (%) |
|  | MCN | MCN |  | MCN | MCNd | MCNp | UN |  |  |  | MCN | MCNd | MCNp | UN |  |  |  |
| 1 | 1.115 | 0.832 | 74.6 | 1.033 | 0.866 | 0.853 | 0 | 83.9 | 98.5 | 0 | 1.209 | 0.714 | 0.449 | 0.161 | 59.1 | 63.0 | 22.6 |
| 2 | 1.453 | 0.669 | 46.1 | 0.952 | 0.427 | 0.384 | 0 | 44.8 | 90.1 | 0 | 1.247 | 0.927 | 0.810 | 0.485 | 74.4 | 87.3 | 52.3 |
| 3 | 0.962 | 1.201 | 124.9 | 1.419 | 1.191 | 1.097 | 0 | 84.0 | 92.1 | 0 | 1.036 | 1.361 | 0.972 | 0.577 | 131.4 | 71.5 | 42.4 |
| 4 | 1.357 | 1.170 | 86.2 | 1.649 | 1.343 | 1.327 | 0 | 81.4 | 98.8 | 0 | 0.676 | 0.636 | 0.625 | 0.405 | 94.0 | 98.3 | 63.7 |
| 5 | 1.256 | 1.055 | 84.0 | 1.004 | 1.088 | 1.179 | 0 | 108.3 | 108.4 | 0 | 1.108 | 1.089 | 0.256 | 0.787 | 98.3 | 23.5 | 72.2 |
| 6 | - | - | - | 1.015 | 1.587 | 1.480 | 0 | 156.4 | 93.3 | 0 | 1.196 | 1.023 | 0.180 | 1.045 | 85.6 | 17.6 | 102.1 |

**Table 2b – MT (N)**

| **Animal** | **Group A** | | | **Group B** | | | | | | | **Group C** | | | | | | |
| --- | --- | --- | --- | --- | --- | --- | --- | --- | --- | --- | --- | --- | --- | --- | --- | --- | --- |
|  | **MT** | | | **MT** | | | | | | | **MT** | | | | | | |
|  | Left | Right | RI (%) | Left | Right | | | RI (%) | CR_MCNp_ (%) | CR_UN_ (%) | Left | Right | | | RI (%) | CR_MCNp_ (%) | CR_UN_ (%) |
|  | MCN | MCN |  | MCN | MCNd | MCNp | UN |  |  |  | MCN | MCNd | MCNp | UN |  |  |  |
| 1 | 0.448 | 0.270 | 60.3 | 0.411 | 0.351 | 0.334 | 0 | 85.4 | 95.3 | 0 | 0.495 | 0.205 | 0.130 | 0.038 | 41.5 | 63.1 | 18.6 |
| 2 | 0.605 | 0.231 | 38.2 | 0.416 | 0.145 | 0.141 | 0 | 35.0 | 97.2 | 0 | 0.532 | 0.367 | 0.329 | 0.161 | 69.0 | 89.6 | 43.8 |
| 3 | 0.451 | 0.526 | 116.7 | 0.569 | 0.493 | 0.463 | 0 | 86.7 | 93.9 | 0 | 0.518 | 0.581 | 0.438 | 0.236 | 112.0 | 75.4 | 40.7 |
| 4 | 0.504 | 0.444 | 88.0 | 0.613 | 0.632 | 0.613 | 0 | 103.1 | 96.9 | 0 | 0.305 | 0.198 | 0.190 | 0.122 | 65.0 | 96.0 | 61.6 |
| 5 | 0.466 | 0.405 | 86.9 | 0.415 | 0.500 | 0.508 | 0 | 120.3 | 101.6 | 0 | 0.497 | 0.409 | 0.082 | 0.293 | 82.4 | 20.0 | 71.6 |
| 6 | - | - | - | 0.471 | 0.642 | 0.616 | 0 | 136.3 | 96.0 | 0 | 0.539 | 0.386 | 0.048 | 0.370 | 71.5 | 12.4 | 95.9 |

**Table 2c – EMG PTP (mV)**

| Animal | **Group A** | | | **Group B** | | | | | | | **Group C** | | | | | | |
| --- | --- | --- | --- | --- | --- | --- | --- | --- | --- | --- | --- | --- | --- | --- | --- | --- | --- |
|  | **EMG PTP** | | | **EMG PTP** | | | | | | | **EMG PTP** | | | | | | |
|  | Left | Right | RI (%) | Left | Right | | | RI (%) | CR_MCNp_ (%) | CR_UN_ (%) | Left | Right | | | RI (%) | CR_MCNp_ (%) | CR_UN_ (%) |
|  | MCN | MCN |  | MCN | MCNd | MCNp | UN |  |  |  | MCN | MCNd | MCNp | UN |  |  |  |
| 1 | 14.7 | 4.1 | 27.9 | 6.7 | 8.3 | 7.9 | 0 | 123.9 | 95.2 | 0 | 5.9 | 1.9 | 1.4 | 0.3 | 32.2 | 73.7 | 15.8 |
| 2 | 14.7 | 5.4 | 36.7 | 11.5 | 4.9 | 3.3 | 0 | 42.6 | 67.3 | 0 | 2.1 | 33.9 | 31.9 | 6.2 | 1614.3 | 94.1 | 18.3 |
| 3 | 35.5 | 22.6 | 63.7 | 9.9 | 24.2 | 23.4 | 0 | 244.4 | 96.7 | 0 | 25.5 | 29.0 | 28.2 | 8.1 | 113.7 | 97.2 | 27.9 |
| 4 | 14.8 | 13.3 | 89.9 | 29.2 | 15.3 | 15.2 | 0 | 52.4 | 99.3 | 0 | 6.4 | 10.4 | 10.5 | 7.7 | 162.5 | 101.0 | 74.0 |
| 5 | 31.9 | 10.6 | 33.2 | 3.9 | 6.8 | 6.8 | 0 | 174.4 | 100.0 | 0 | 17.7 | 16.3 | 1.5 | 10.1 | 92.1 | 9.2 | 62.0 |
| 6 | - | - | - | 12.7 | 25.4 | 24.4 | 0 | 204.8 | 96.1 | 0 | 20.8 | 13.1 | 2.4 | 13.7 | 63.0 | 18.3 | 104.6 |

**Table 3a Cell count**

| **Animals** | **Native** | | **Group A** | **Group B (nt)** | | **Group C (nt)** | |
| --- | --- | --- | --- | --- | --- | --- | --- |
|  | **MCN** | **UN** | **MCN** | **MCN** | **UN** | **MCN** | **UN** |
| 1 | 151 | 153 | 143 | 176 | 67 | 167 | 106 |
| 2 | 154 | 166 | 134 | 182 | 96 | 124 | 96 |
| 3 | 158 | 184 | - | - | - | - | - |
| 4 | 168 | 146 | - | - | - | - | - |
